# The timing of fireworks-caused wildfire ignitions during the 4^th^ of July holiday season

**DOI:** 10.1371/journal.pone.0291026

**Published:** 2023-09-01

**Authors:** Richard S. Vachula, Jake R. Nelson, Anthony G. Hall

**Affiliations:** Department of Geosciences, Auburn University, Auburn, Alabama, United States of America; University of Ferrara, ITALY

## Abstract

Although anthropogenic climate change is causing increased wildfire activity in the United States (US), humans are also an important ignition source. Humans cause a surge in wildfire ignitions every 4^th^ of July (Independence Day in the US) through the use of fireworks. We examine the 4^th^ of July peak in fireworks-caused wildfire ignitions and show that their spatial distribution varies but has been heavily concentrated in the west and north central US and predominantly on tribal lands. Further, we show that the weekly timing of the 4^th^ of July influences both the number and weekly distribution structure of fireworks-caused ignitions. We interpret these weekly and daily-scale distribution patterns of fireworks-caused ignitions to reflect the influences of human behavioral variations, culture, and fireworks regulations. For example, our analysis suggests that weekends and religious days of rest (e.g., Saturday, Sunday) have a dampening effect on the number on wildfire ignitions due to fireworks, and that weekends and the timing of work holidays likely impact the weekly distribution of fireworks-caused ignitions. Additionally, comparisons of fireworks-caused ignitions before and after the 4^th^ of July at the daily and weekly scale likely reflect the efficacy of firework sales regulations and human behavioral tendencies towards pre-holiday impulsiveness. Given the predictability of the fireworks-caused ignitions and rising costs of wildfire mitigation, these results have several important management and policy implications.

## Introduction

Recent fire seasons in the United States (US) have been particularly alarming [1]. Although anthropogenic climate change is partly responsible for this increased fire activity [2, 3], land management techniques and their historical legacies also influence landscape flammability [4, 5]. The role of humans on modern fire activity is complex, as they can directly cause fire ignitions while also dampening the impacts of climate change on fire [6, 7].

Humans cause wildfire ignitions in numerous ways [8]. Some human-caused ignitions are due to more structural components of modern society like powerlines, roads, and railroads [9–11]. In contrast, other ignition sources are more attributable to human behaviors, such as smoking, burning trash, and arson [12, 13]. Regardless of the means by which humans cause wildland fire ignitions, they are having a marked impact and now dominate fire activity in the US [6]. In light of the rising costs of wildfire management in the US [14], a more thorough understanding of how humans cause wildfires is increasingly important.

**Fig 1:**
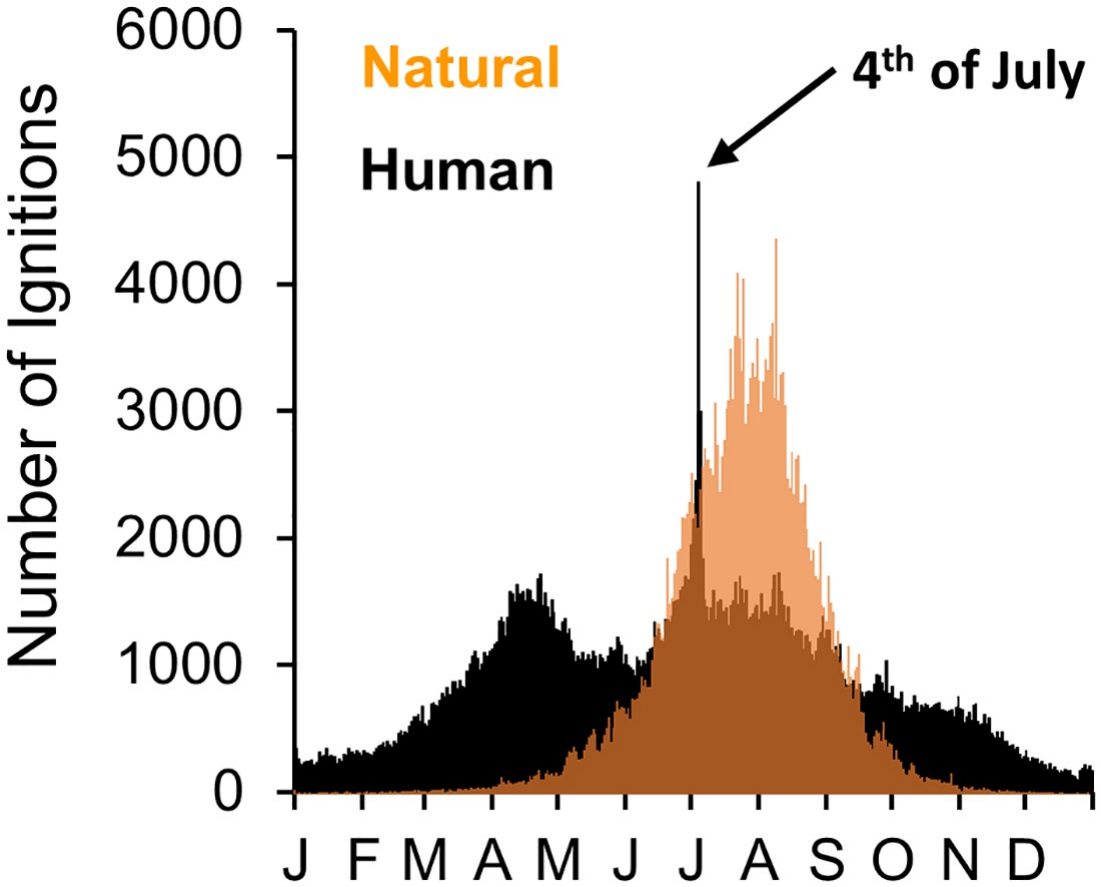
Annual wildland fire ignitions. The annual distribution of natural (orange) and human-caused (black) wildland fire ignitions in the US (data from the Federal Wildland Fire Database; 1980–2016 CE).

Fireworks are one of the more fascinating ways in which humans cause wildfire ignitions in the US. Fireworks on Independence Day (4^th^ of July) are known to cause a precipitous increase of wildfire ignitions in the US [6, 15], which is particularly striking in the context of the calendar year (Fig 1). Fireworks are a hallowed component of Independence Day celebrations, despite their negative effects on air quality [16, 17] and the risk of personal injury that they pose [18]. Although the holiday-induced increase of fireworks-caused wildfire ignitions has been noted and described in previous research [6, 15], there has not been a thorough examination of this aspect of American culture and human-environment interactions.

The analysis of weekly cycles has been a successful approach to characterizing how human culture impacts fire activity. Previous work focusing on fire in Africa has shown that religious affiliation is closely associated with the weekly timing of burning in croplands, with decreases of fire during religious days of rest (e.g., Sunday and Friday) [19]. Likewise, a global analysis showed that weekly fire cycles are generally associated with the structure of workweeks [20]. In the US, fires tend to follow the workweek, with decreases concentrated during weekends (Saturday and Sunday) [20].

The policies and laws governing the use of fireworks at both the consumer and commercial level vary across the US. The Bureau of Alcohol, Tobacco, Firearms, and Explosives (ATF) is the federal-level entity that manages the legality of fireworks. Generally, the ATF governs the importing, manufacturing, and dealing of commercial fireworks that are most often seen during large public-events (e.g., a city- or county-sponsored firework show). The use of commercial fireworks requires a Federal explosive license [21] or other permits to operate. The National Fire Protection Association (NFPA) also maintains codes that set minimum safety standards for commercial fireworks operation [22], including that operators have many hours of training for the safe and responsible use of fireworks.

Consumer fireworks, on the other hand, are not regulated by the ATF, however, the type of fireworks available to consumers must meet Consumer Product Safety Commission (CPSC) codes. This category of fireworks is smaller than display fireworks and commonly found at stands throughout the US, which may operate annually or seasonally (around the 4^th^ of July). The specifics of consumer firework sales and their public use are left to states, counties, or city governments to decide with guidance and use-limitations following the CPSC codes. As a result, there is both inter- and intrastate variation in firework policies. For example, Illinois prohibits the use of nearly all fireworks whereas Wyoming allows the sale of any consumer firework that meets CPSC guidelines and requirements. The minimum age to buy fireworks also varies by state with most requiring consumers to be at least 16 but others allowing purchase by children as young as 12 (e.g., Oklahoma and North Dakota).

Complicating matters further, many state-level firework regulations leave room for local municipalities to enforce stricter rules including where, when, and what fireworks can be bought and used by consumers. For example, some cities that allow firework sales do so only for the weeks leading up to the 4^th^ of July (i.e., [23]). Additionally, even if a state has an outright ban on the sale of some fireworks, there may still be areas where they can be bought and sold to consumers, such as on Native American tribal lands [24].

Here we examine the Independence Day (4^th^ of July) peak in fireworks-caused ignitions of wildfires in more detail. We aim to understand how these fire ignitions reflect human culture, behavior, and policy in the US. Although fireworks obviously cause increases of wildfire ignitions (Fig 1), how these ignitions vary interannually and in response to human behaviors is not well understood. Through the lens of weekly cycle analyses, we investigate how the weekly timing of the 4^th^ of July influences both the number and weekly distribution of wildfire ignitions attributed to fireworks.

## Data and methods

We analyzed the Federal Wildland Fire Occurrence dataset compiled by the US Geological Survey (USGS) using data from numerous agencies. This dataset was downloaded and archived by the authors on June 21, 2017. The hosting of this wildland fire data by the USGS was discontinued in 2020 and transferred to the National Interagency Fire Center (https://www.nifc.gov/). The dataset details nearly 600,000 fires that burned on federal lands in the US between 1980 and 2016. Although similar datasets spanning more recent years are available [25, 26], this dataset provides the longest historical record of the specific causes of wildland fire ignitions. From this dataset, we extracted fires with causes attributed to fireworks which occurred between June 28 and July 11 each year (one week prior to and after the 4th of July; n = 7068). We consolidated these data by generating time series of total daily ignitions attributed to fireworks. Concurrently, we determined the day of the week on which the 4^th^ of July fell each year to group these data and time series. To generate mutually comparable two-week period time series, we normalized them by calculating the daily ignitions as a relative percentage of the number of ignitions each 4^th^ of July. We also compare the ignition data to fireworks consumption data which were downloaded from the American Pyrotechnics Association (https://www.americanpyro.com/industry-facts-figures), but compiling data from the US Department of Commerce and US International Trade Commission.

The latitude and longitude information associated with each ignition was used to geolocate the event and subsequently transformed into a vector shapefile. We used a combination of land designation areas provided by ESRI (National and State parks and forests, County, Regional and Local parks), the Protected Areas Database (PAD) of the US (including Bureau of Land Management areas), and US Census TIGER line files (Native American Reservation boundaries) to determine the land type on which each ignition took place [27, 28]. For each ignition, an attribute was added that indicated whether the ignition was on public land, tribal land, federally managed land on tribal land, or private land. Where the latter is concerned, we assumed that any ignition that did not fall within the bounds of the land designation areas took place on private land. The methodology and workflow followed in this study is summarized in S2 Fig.

## Results

**Fig 2:**
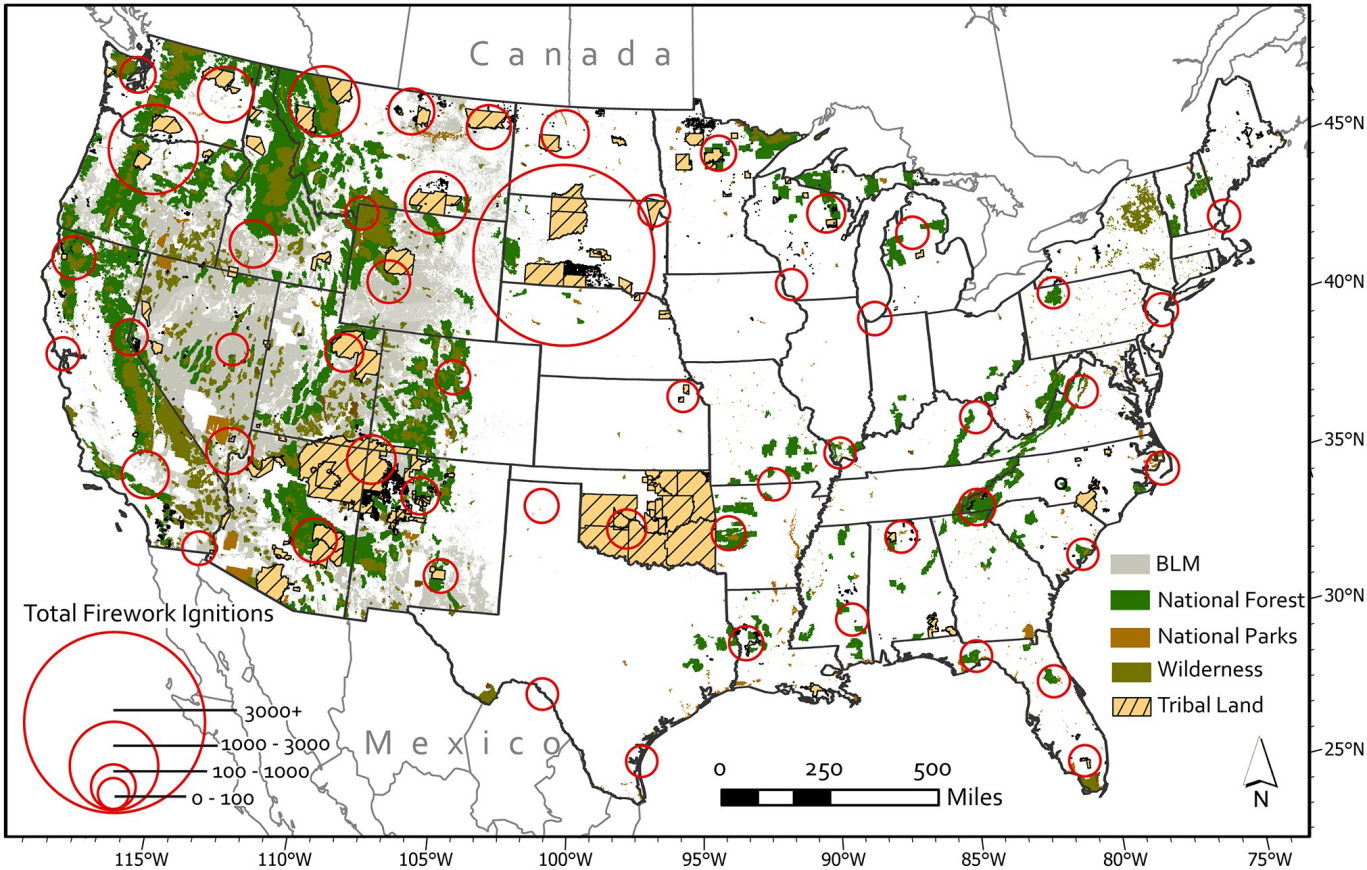
Map depicting hotspots of fireworks-caused wildfire ignitions and how they correspond to land types. Mapped land types include areas managed by the Bureau of Land Management (BLM), National Forest, National Parks, Tribal Land, and Wilderness area. Select land use designations and boundaries were collected from the United States Geologic Survey Protected Areas Database 3.0 [28]. Tribal land boundaries were collected from the US Census Tigerline Shapefile database [29].

**Fig 3:**
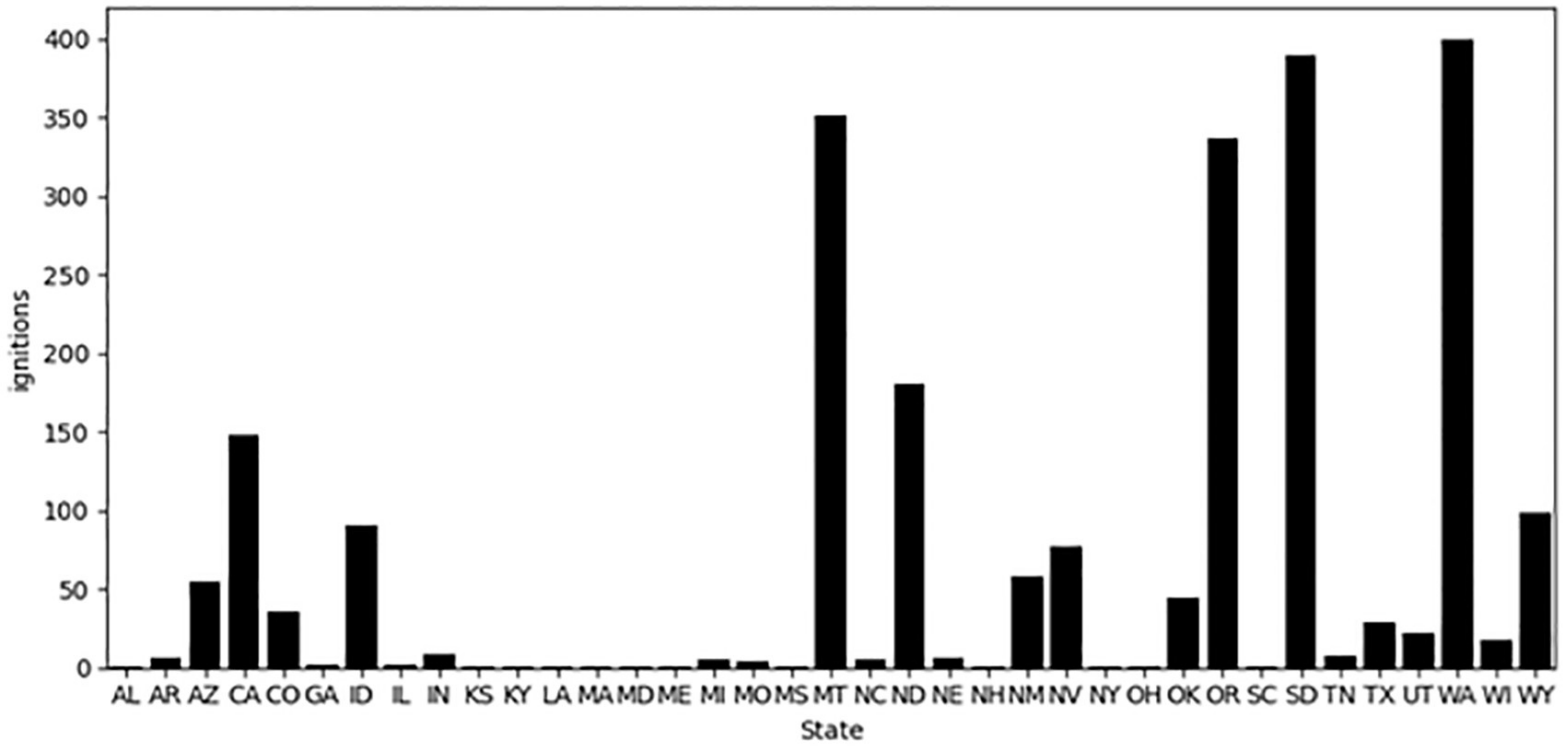
Total number of fireworks-caused wildfire ignitions by state.

Over the 37-year period we analyzed (1980–2016 CE), a total of 11,294 wildland fire ignitions were attributed to fireworks. Of these, the bulk (69.7%) were situated on Native American tribal lands, whereas relatively fewer occurred on public lands (24.6%), privately owned lands (5%), and federally managed areas on tribal land (0.7%). The spatial distribution of ignitions and the boundaries of the largest land use designation types is illustrated in Fig 2. Rather than display each of the 11,294 ignitions individually, we have aggregated them as a method of cartographic generalization. Ignition points are assigned to a cluster based on their location and distance to the cluster center, while the location of the cluster representation (red circle) is based on the overall distribution of ignitions and the number of data bins. Important to keep in mind is that the ignitions represented by each circle will not necessarily fall within the bounds of the circle. Rather, it provides a geographic approximation of the corresponding ignitions center of mass. This visualization approach allows one to evaluate the general location of ignition sources in a region while also allowing for the interrogation of corresponding land uses in the area. On a regional scale, we see that the number of firework ignitions is decidedly concentrated in the western (W) and north central (NC) US, and particularly so for Washington, Oregon, Montana, and the Dakotas where the red circles are largest (Fig 3).

Nearly two-thirds (62.6%) of the fire ignitions attributed to fireworks in the database occurred during the two-week period encompassing the 4th of July (June 28^th^ to July 11^th^). Of these ignitions, the same general proportion of ignitions occurred on tribal land (76.8%), public lands (17.9%), privately owned lands (4.6%), and federally managed areas on tribal land (0.8%). From 1980 to 2016, the 4^th^ of July fell on Monday, Friday, and Saturday six times each, whereas it fell on Sunday, Wednesday, and Thursday five times each. There were only four 4^th^ of Julys that fell on Tuesdays.

**Fig 4:**
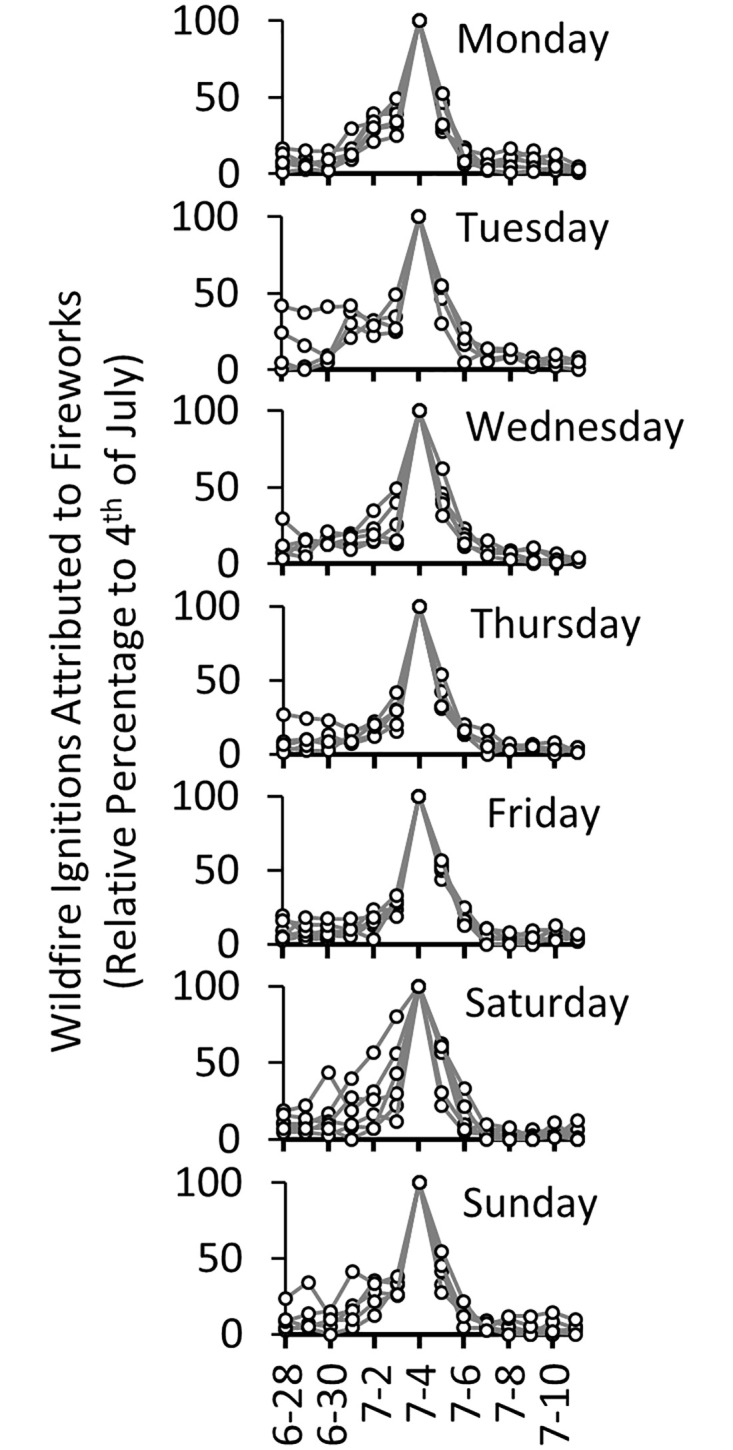
Daily fire ignitions attributed to fireworks for the two-week period encompassing Independence Day. The two-week periods (June 28 to July 11) are grouped by the day of the week on which Independence Day falls (4^th^ of July). The number of fire ignitions are normalized as relative percentages of the number of ignitions occurring on each 4^th^ of July.

Regardless of the day of the week on which the 4^th^ of July fell, a clear peak in fireworks-caused ignitions occurred on the holiday each year. When normalized as a relative percentage of the total fireworks-caused ignitions that occurred on the 4^th^, a persistent weekly scale cycle of fireworks-caused ignitions is evident (Fig 4). The number of wildfire ignitions attributed to fireworks was generally lower when the 4^th^ of July fell on a Saturday or Sunday, relative to when it fell on weekdays (S1 Fig). This was also the case when considering the number of fireworks-caused ignitions over the two-week period encompassing the 4^th^ of July (S1 Fig). Although there was considerable variability of ranges and distributions of fireworks ignition numbers between days of the week on which the 4^th^ of July fell, ANOVA (analysis of variance) tests cannot reject the null hypothesis that the means of the distributions are equal (for both 0.05 and 0.1 significance levels) for both the 4^th^ of July and the two-week period encompassing it (S1 Fig). In other words, annual numbers of firework ignitions were not significantly different when grouped by the day of the week in which the 4^th^ of July fell.

**Fig 5:**
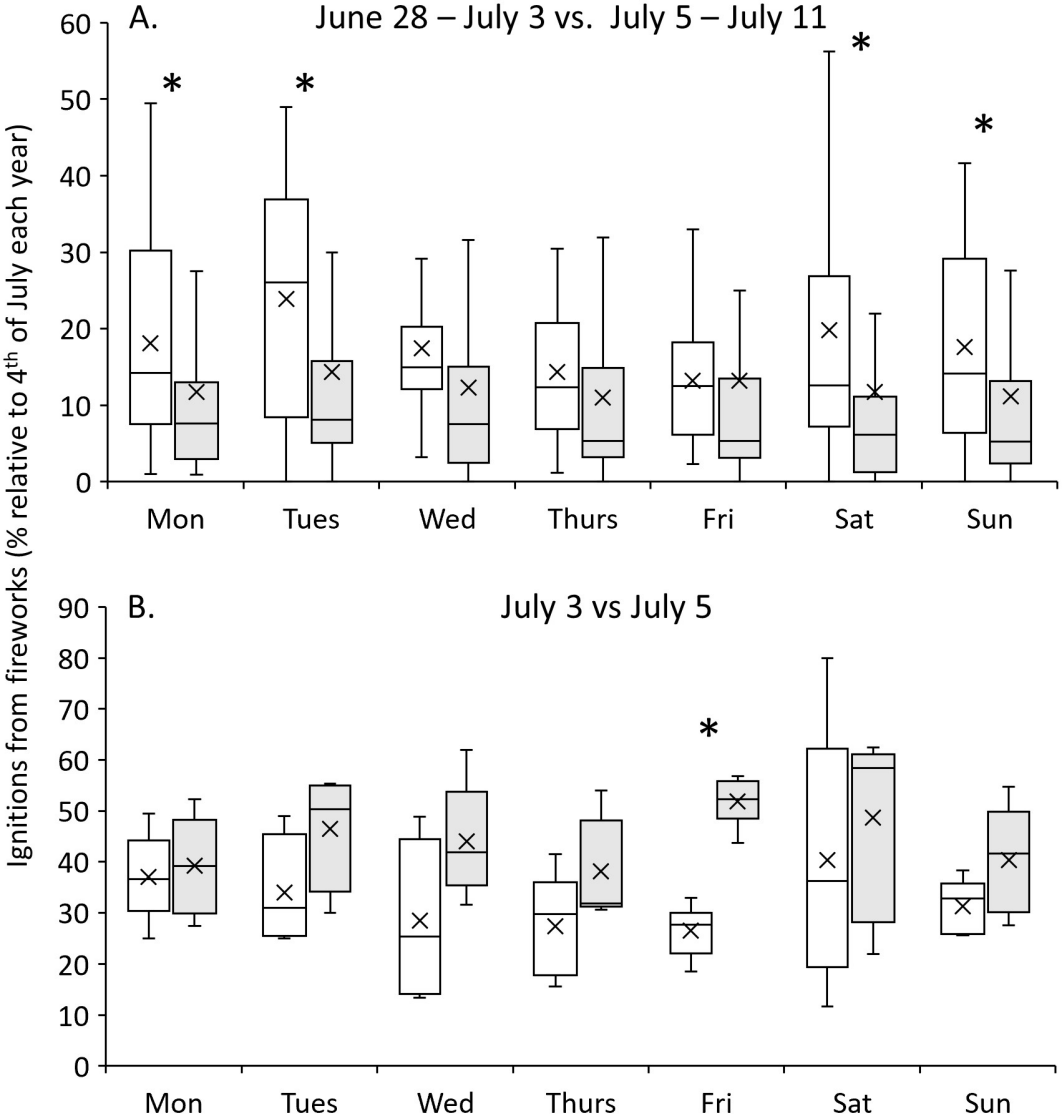
Comparison of the fire ignitions from fireworks before and after the 4^th^ of July as a function of the day of the week on which the holiday fell. For each year, each two-week time series was normalized as a relative percentage of ignitions on the 4^th^,. A) Comparison of ignitions due to fireworks the week prior to the 4^th^ of July (white; June 28 to July 3), relative to the week after (gray; July 5 to 11). B) Comparison of ignitions due to fireworks the day after the 4^th^ of July (gray; July 5) relative to the day before it (white; July 3). Boxes depict the median, and upper (75^th^ percentile) and lower (25^th^ percentile) quartiles. Whiskers depict the minimum and maximum. The x markers illustrate the mean of each distribution. Asterisks denote significantly different means between the before/after distributions, as per t-tests assuming unequal variances and two-tailed distributions (p < 0.05).

That said, the relative distribution of daily wildfire ignitions due to fireworks during the two-week period encompassing the 4^th^ of July varied depending on the day of the week on which the 4^th^ of July fell (Fig 4). When the 4^th^ of July fell on a day immediately following a weekend (e.g., Monday, Tuesday), a shoulder of fireworks-caused ignitions occurred over the preceding weekend. In contrast, when a weekend immediately followed the 4^th^ of July (e.g., when the 4^th^ fell on a Thursday or Friday), the uptick in firework ignitions was not as pronounced. When the 4^th^ of July fell on a weekend (e.g., Saturday or Sunday), considerable numbers of fireworks-caused ignitions occurred during the week preceding the holiday (especially so for a Saturday 4^th^ of July), whereas they tapered off the following week. These patterns are perhaps more visible when considering the weeks before and after the 4^th^ of July in aggregate (Fig 5A). Indeed, the mean relative number of ignitions in the week before and after the 4^th^ of July are significantly different when the 4^th^ falls on a Monday, Tuesday, Saturday, and Sunday, as per t-test results (p < 0.05). Regardless of t-test results, for every day on which the 4^th^ of July fell, the mean and median relative ignition numbers decreased from the week preceding the 4^th^ relative to the week following the 4^th^.

In contrast to the before vs. after the 4^th^ of July differences exhibited on the weekly timescale, the opposite trend is exhibited on the daily timescale. For each day of the week on which the 4^th^ of July fell, there was a general tendency for the 5^th^ of July to experience more fireworks-caused ignitions than the 3^rd^ of July (Fig 5B). Although the mean relative number of ignitions was only significantly different the day before and after the 4^th^ of July when it fell on a Friday, both the medians and means were greater for the day after the 4^th^ relative to the day before it for every day of the week on which the 4^th^ fell. More broadly, during the two week period encompassing the 4^th^, 34.2% of fireworks-caused ignitions occurred on the 4^th^, whereas 10.5% and 14.9% occurred on the 3^rd^ and 5^th^, respectively.

**Fig 6:**
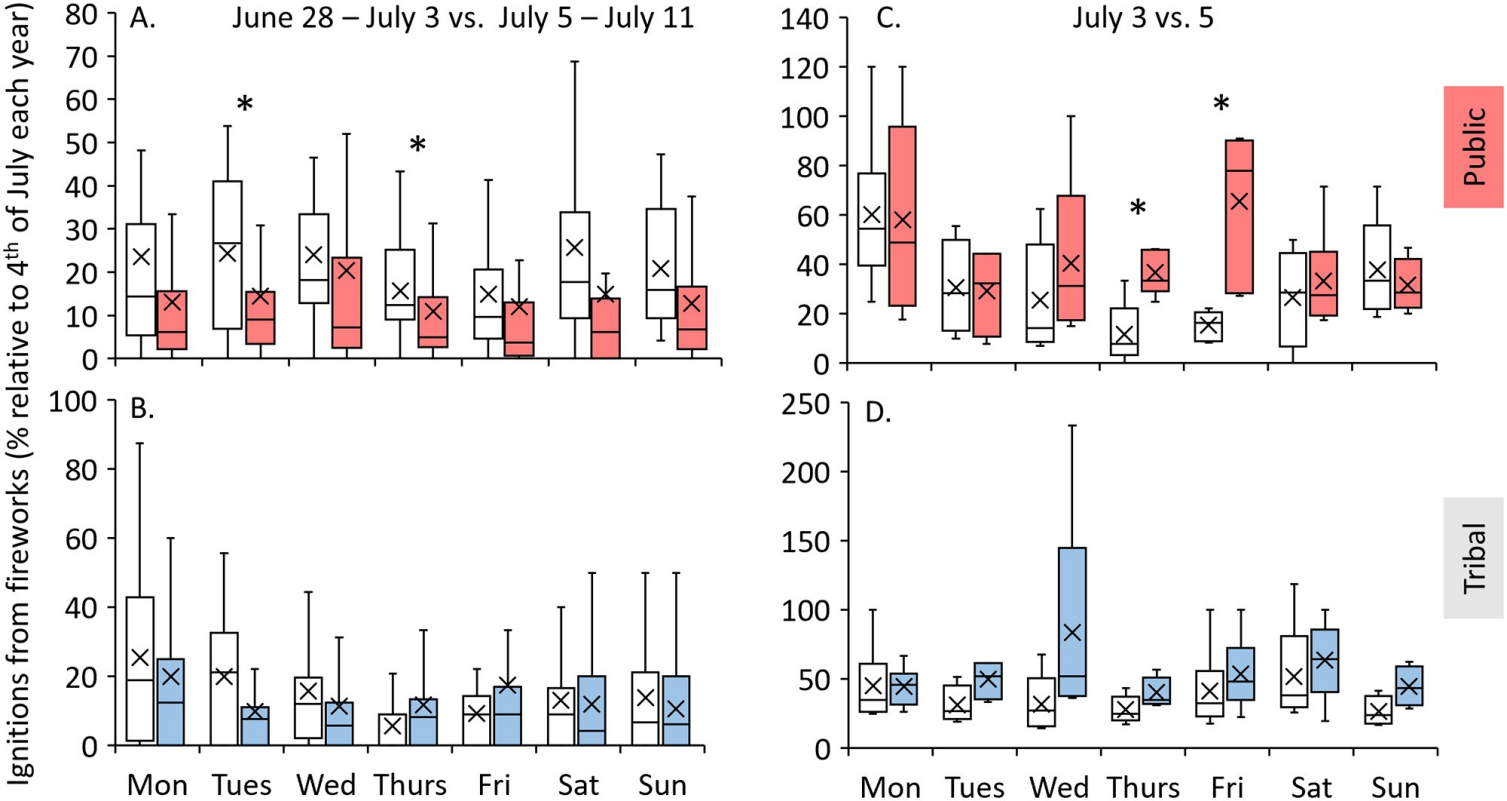
Comparison of timing of wildfire ignitions from fireworks on public and Native American tribal lands. Wildfire ignitions from fireworks on public lands (A, C) and Native American tribal lands (B, D) the weeks (A, B) and days (C, D) before (white) and after (red and blue) the 4^th^ of July (normalized as a relative percentage of ignitions on the 4^th^ each year), as a function of the day of the week on which the 4^th^ of July fell. Boxes depict the median, and upper (75^th^ percentile) and lower (25^th^ percentile) quartiles. Whiskers depict the minimum and maximum. The x markers illustrate the mean of each distribution. Asterisks denote significantly different means between the before/after distributions, as per t-tests assuming unequal variances and two-tailed distributions (p < 0.05).

Breaking the temporal patterns down by land category reveals further variability in the patterns of before vs. after the 4th of July, especially differences on public lands and Native American tribal lands, respectively (Fig 6). On the weekly timescale, the trend of more ignitions due to fireworks the week prior to the 4^th^ of July relative to the week after was evident on public lands for each day of the week on which the 4^th^ fell (Fig 6A). In contrast, on Native American tribal lands (Fig 6B) there were only more fireworks-caused ignitions during the week prior to the 4^th^ if it fell on a Monday, Tuesday, or Wednesday, although the difference is not statistically significant. When the 4^th^ fell on a Friday, Saturday, or Sunday, fireworks-caused ignitions the week before and after the 4^th^ were nearly equivalent on tribal lands (Fig 6B). On the daily timescale, there were consistently more ignitions on tribal land due to fireworks the day after the 4^th^ of July relative to the day before it (Fig 6D). In contrast, there were generally more fireworks-caused ignitions on public lands the day after the 4^th^ relative to the day before it when the 4^th^ fell on a Wednesday, Thursday, and Friday, but not when it fell on other days of the week (Fig 6C).

## Discussion

Using the Federal Wildland Fire Occurrence dataset we explore the spatial and temporal patterns of the wildfire ignitions attributed to fireworks. As one might expect, the number of firework-caused ignitions is substantially higher on the 4^th^ of July than at any other time of the year. In addition, our analysis of the number of ignitions in the weeks and days leading up to and following the 4^th^ of July reveals a notable uptick (or shoulder period) in fireworks caused ignitions when compared to the rest of the year. Further analysis revealed that this pre- and post-4^th^ of July uptick varies as a function of the day of the week on which the 4th falls. Moreover, the spatial distribution of the firework ignitions also varies by region but has been heavily concentrated in the west and north central US and predominantly on tribal land. Given the recent increase in major wildfires across the western US, these results have several important management and policy implications worth further discussion.

### Weekly cycles of fireworks-caused wildfire ignitions

Our analyses show that the daily distribution of fireworks-caused ignitions during the 4^th^ of July holiday season varies as a function of the day of the week on which the 4^th^ falls. Although the total number of wildfire ignitions attributed to fireworks varies depending on the 4^th^’s day of the week, these differences are not necessarily meaningful in a purely statistical sense. That said, we found that the numbers of total ignitions were generally lower (as demonstrated by lower median and mean values) when the 4th of July fell on a weekend (Saturday or Sunday), relative to when it fell on weekdays (S1 Fig). This is surprising. Although some previous research has demonstrated that weekends and religious days of rest are associated with decreased wildfire ignitions [19, 20], other work has shown fireworks use causes increased numbers of ignitions on weekends, public holidays, and during cultural events [30, 31]. Our analyses therefore suggest that the weekend dampening effect on ignitions imprints upon the holiday heightening of ignitions numbers due to fireworks.

One complicating factor in the analysis of weekly ignition cycles during the Independence Day season is that the timing of federal and state holidays (days off from work for many, but certainly not all) does not necessarily coincide with the actual 4^th^ of July. For example, if the 4^th^ of July falls on a Saturday or Sunday, the holiday is typically granted on Friday or Monday, respectively, giving some working people a 3-day weekend. Conversely, if the 4^th^ of July falls during the week (e.g., Tuesday, Wednesday, Thursday), then any day-off would be independent of the weekend. The presence of a shoulder of fireworks-caused ignitions on the weekend preceding 4^th^ of Julys that fell on a Monday or Tuesday (Fig 4) illustrates the impacts of days off from work on the weekly distribution of fireworks-caused ignitions.

Generally, we found that there were more fireworks-caused ignitions the week before the 4^th^ of July relative to the week after it. This was particularly true for the years when the 4^th^ of July fell on a Saturday and Sunday (also the days with the lowest 4^th^ of July ignitions overall), suggesting that the dampening effect of the 4^th^ of July falling on a weekend may have a certain “spillover” that increases consumers’ willingness to begin their celebrations early. That firework ignitions were higher the week before the 4th may also reflect several other factors such as the state and local policies that determine when fireworks can be legally bought and used. In many states, there is a window in which this commerce can happen. For example, in the State of Oregon, firework sales can only take place between June 26^th^ and July 6^th^, which coincides with the buildup in firework related ignitions prior to the 4th, and the drop off in ignitions in the following week (Fig 5). Additionally, there is the potential that the pre-4^th^ of July ignition build-up reflects human behavioral tendencies towards impulsivity referred to as “holiday euphoria” [32]. In contrast, evidence from stock market returns suggests a tendency towards less risky investor behavior leading up to holidays [33, 34].

At the daily scale, our analyses show that wildfire ignitions due to fireworks were greater the day after the 4^th^ of July relative to the day before it (Fig 5B). Indeed, during the two-week period encompassing the 4^th^, the 4^th^ accounted for 34.2% of fireworks-caused ignitions whereas 10.5% and 14.9% occurred on the 3^rd^ and 5^th^, respectively. This is potentially an artefact of a data reporting lag (e.g., a fire started at 11 pm but not identified or reported until the following day). However, it is difficult to ascertain whether this could explain the consistency evident in the data. Alternatively, the greater ignitions occurring the day after the 4^th^ relative to the day before it could also reflect both policies and market forces surrounding fireworks sales and subsequent fireworks-caused ignitions. The elevated fireworks-caused ignitions on the 5^th^ could reflect peoples’ choices to consume fireworks for the sake of being rid of them as many ordinances allowing fireworks sales and use elapse on the 5^th^, leaving people with the choice of using them or needing to store them until the following year. Likewise, if sales are restricted to this time window, sellers may cut prices to offload excess supply, leading to a rush of fireworks purchasing and more fire ignitions as their use becomes more careless.

Public and tribal lands exhibit differences in the relative numbers of fireworks-caused ignitions before and after the 4^th^ of July at the weekly scale, which supports our inferences regarding the effectiveness of fireworks regulation policies in controlling these distributions. On the weekly scale, the before vs. after 4^th^ of July pattern is more strongly pronounced on public lands relative to tribal lands. We interpret this relationship to reflect the differences of fireworks regulations on public and tribal lands; regulations tend to be more stringent on public lands relative to tribal lands (where the same regulations that apply to public lands may or may not apply depending on their nature [35]). Given that many regulations limit the timing of fireworks sales to the weeks prior to the 4^th^ of July (i.e., [23]), we interpret the stronger pattern of before vs. after 4^th^ of July signal on public lands to reflect the efficacy of these policies in limiting fireworks sales and subsequent wildfire ignitions.

### Policy and management implications

The annual 4^th^ of July peak in wildfire ignitions represents a predictable event for which firefighters could plan, but staffing and resource guidance is not structured in a way that facilitates this planning. The National Fire Protection Association (NFPA) is a nonprofit organization that publishes codes to guide career and volunteer fire departments in daily operations, staffing, and resource guidance [36, 37]. Although not public policy in the strictest sense, the NFPA provides industry standards that illustrate how fire departments should operate regardless of localized policy variations. The NFPA codes provide guidance for minimum staffing requirements for fire departments, but these guidelines are primarily based on temporally static characteristics of the jurisdiction in question (e.g., number of fire department stations, population and population density, landscape characteristics), the personnel needs of equipment (e.g., the number of firefighters needed to operate a ladder truck), and the remoteness and size of a fire [36, 37]. Further, there is a specific code for planning for wildfire responses, but it mostly focuses on preparedness, financial planning, safety, and personnel training, as opposed to staffing or scheduling guidelines [38]. Our analyses show that historical records of wildfire ignition dates and causes can yield information relevant to firefighter staffing and scheduling, highlighting a gap in the NFPA standards. Given the predictability of fireworks caused ignitions of wildfires, we therefore suggest that data-driven approaches be integrated into NFPA codes as well as fire department policies to ensure adequate staff scheduling to deal with fireworks-caused wildfires during the 4^th^ of July season.

**Fig 7:**
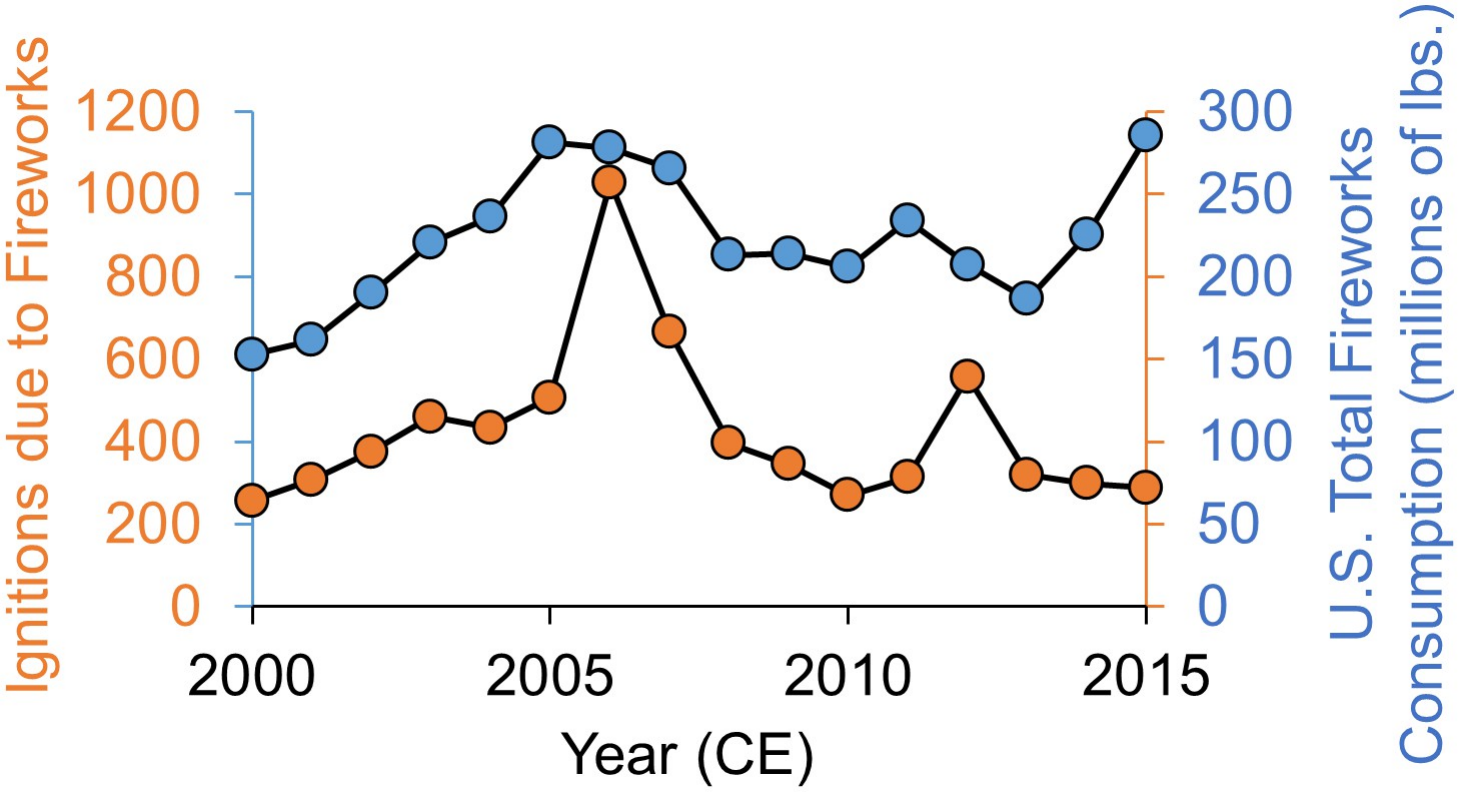
Comparison of annual fireworks-caused wildfire ignitions and fireworks consumption. The number of wildfire ignitions due to fireworks (orange) is positively correlated (r = 0.52, p = 0.03) with the total consumption of fireworks in the U.S. (blue).

More broadly, the annual peak of firework-caused wildfire ignitions is a predictable human impact on the environment that should be the subject of more policy and management discourse. Between 1980 and 2016 CE, more than 11,000 wildland fires were ignited by fireworks, and nearly two-thirds of these ignitions (62.6%) occurred during the two-week period encompassing the 4^th^ of July. Despite any spatial or temporal variability of fire ignitions due to fireworks associated with climate, ecology, fuels, etc., there is a clear positive correlation (r = 0.52, p = 0.03) between the number of wildfire ignitions due to fireworks and consumption of fireworks in the US (Fig 7). Given the rising costs of wildfire management and suppression [14], curtailing this ignition source seems a very actionable means of reducing costs. Although some jurisdictions limit fireworks sales, fireworks regulations are very spatially heterogenous in the US, depending on local and state legislation [39]. Further, depending on the nature of individual state laws, they may or may not apply on adjacent Native American tribal lands [35], complicating the policy landscape. Notwithstanding direct prohibition of fireworks sales, other creative approaches to limiting the impacts of fireworks-caused ignitions may be found in consumer education [39] or by the regulation of fireworks impacts to water quality via the Clean Water Act [40]. Alternatively, the use of drones to produce light shows as an alternative to fireworks shows has become increasingly popular and poses little risk for wildfire ignition [41, 42].

## Conclusions

In this paper, we examine the annual 4^th^ of July peak in fireworks-caused wildfire ignitions. Although this peak in ignitions is striking and has been described in previous research, it has not been examined in thorough detail. We find that these ignitions tend to be concentrated on tribal lands in the west and north central US, though they occur nationwide. Additionally, we find that the weekly and daily-scale distribution patterns of these fireworks-caused ignitions varies depending on the weekly timing of the 4^th^ of July holiday. We discuss these patterns and suggest that human culture, behavior, and regulations influence the occurrence of the ignitions. In light of rising wildfire mitigation costs and the predictable nature of the July 4^th^ peak in ignitions, these findings have several actionable policy and management implications.

## Supporting information

S1 Fig Comparison of the number of fireworks-caused wildland fire ignitions as a function of the day of the week on which the 4^th^ of July fell. Boxplots depict the number of wildland fire ignitions attributed to fireworks (A) on the 4th of July and (B) for the two-week period encompassing it (June 28 to July 11), each year between 1990 and 2016 CE. Fire ignitions are grouped by the day of the week on which the 4th of July falls. Boxes depict the median, and upper (75th percentile) and lower (25th percentile) quartiles. Whiskers depict the minimum and maximum. The x markers illustrate the mean of each distribution.
(TIF)

S2 Fig Method and workflow undertaken in this study. (TIF)

S1 Dataset Underlying data used to reach the conclusions drawn in the manuscript. The dataset combines data from the Federal Wildland Fire Database and land designation areas provided by ESRI, the Protected Areas Database (PAD), and US Census TIGER line files.
(XLSX)
