# Supplementary figures and images for: The timing of fireworks-caused wildfire ignitions during the 4th of July holiday season
Source: PLoS One. 2023 Sep 1;18(9):e0291026. doi: 10.1371/journal.pone.0291026 (PMC10473470; doi:10.1371/journal.pone.0291026)

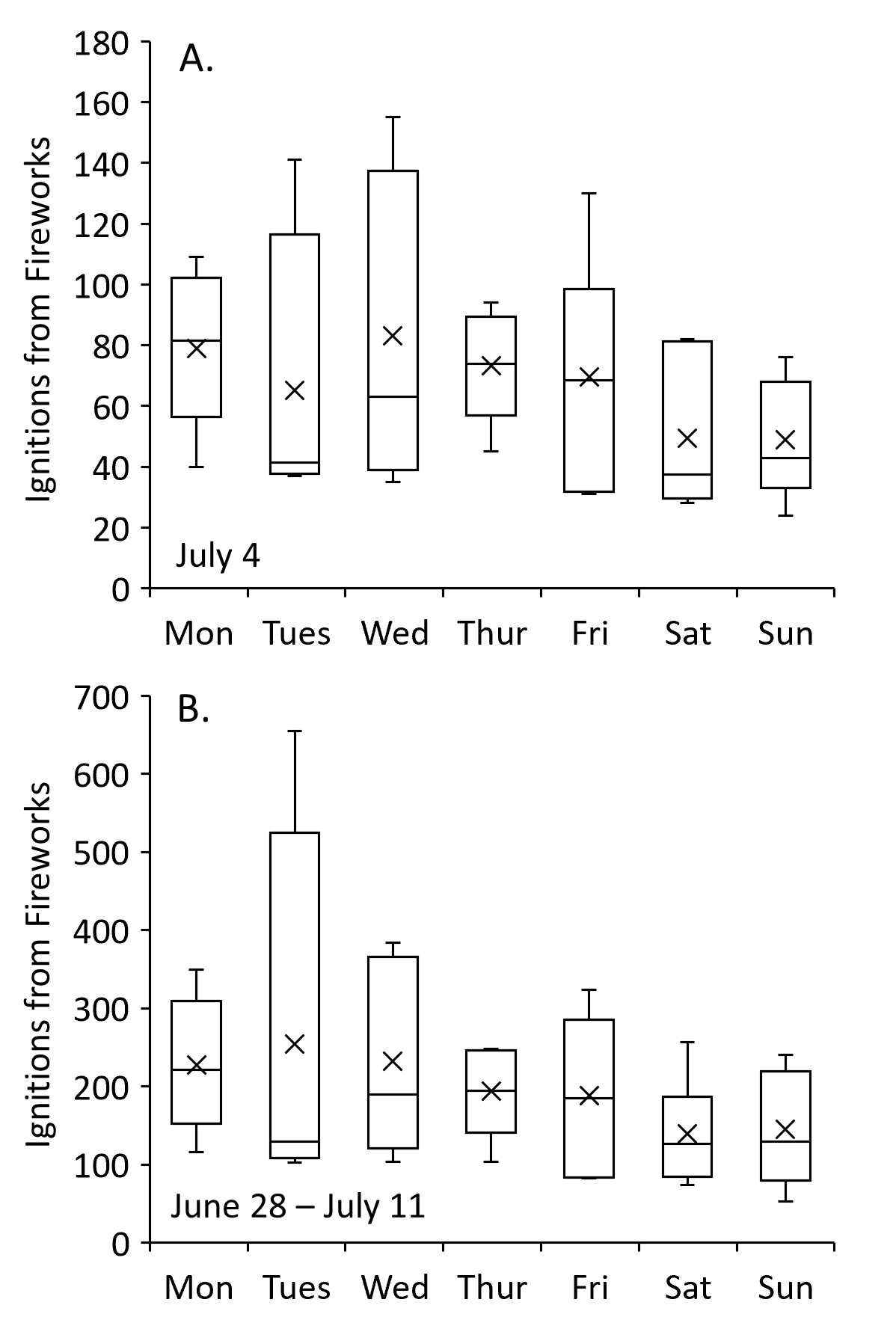

Supplement: S1 Fig — Boxplots depict the number of wildland fire ignitions attributed to fireworks (A) on the 4th of July and (B) for the two-week period encompassing it (June 28 to July 11), each year between 1990 and 2016 CE. Fire ignitions are grouped by the day of the week on which the 4th of July falls. Boxes depict the median, and upper (75th percentile) and lower (25th percentile) quartiles. Whiskers depict the minimum and maximum. The x markers illustrate the mean of each distribution. (TIF) [file pone.0291026.s001.tif]

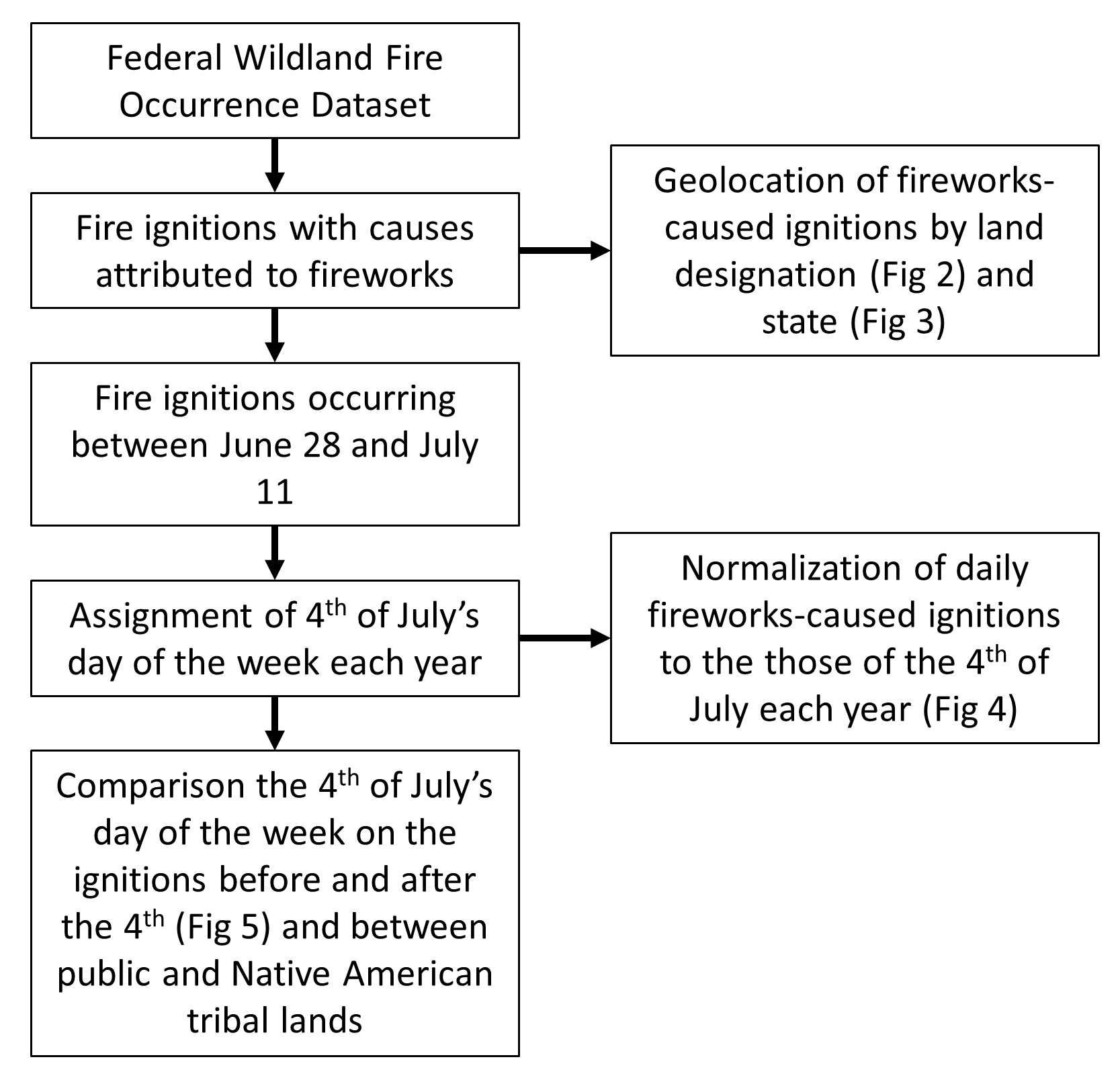

Supplement: S2 Fig — (TIF) [file pone.0291026.s002.tif]
